# The Impact of Controlled Ovarian Stimulation Hormones on the Metabolic State and Endocannabinoid System of Human Cumulus Cells

**DOI:** 10.3390/ijms21197124

**Published:** 2020-09-27

**Authors:** Valentina Notarstefano, Giorgia Gioacchini, Elisabetta Giorgini, Nina Montik, Andrea Ciavattini, Anna Rita Polidori, Fulvia Antonia Candela, Lisa Vaccari, Maurizio Cignitti, Oliana Carnevali

**Affiliations:** 1 Department of Life and Environmental Sciences, Università Politecnica delle Marche, Via Brecce Bianche, 60131 Ancona, Italy; v.notarstefano@univpm.it (V.N.); giorgia.gioacchini@univpm.it (G.G.); e.giorgini@univpm.it (E.G.); 2 Clinica Ostetrica Ginecologica, Università Politecnica delle Marche, Ospedale G. Salesi, Via F. Corridoni 11, 60121 Ancona, Italy; Nina.Montik@ospedaliriuniti.marche.it (N.M.); andrea.ciavattini@ospedaliriuniti.marche.it (A.C.); maurizio.cignitti@ospedaliriuniti.marche.it (M.C.); 3 Centro di Medicina della Riproduzione, Ospedale G. Salesi, Via F. Corridoni 11, 60123 Ancona, Italy; annarita.polidori@ospedaliriuniti.marche.it (A.R.P.); fulviaantonia.candela@ospedaliriuniti.marche.it (F.A.C.); 4 Elettra Sincrotrone Trieste, SISSI Beamline, 34149 Basovizza, Trieste, Italy; lisa.vaccari@elettra.eu

**Keywords:** endocannabinoids, lipid and carbohydrate metabolism, Fourier Transform InfraRed Microspectroscopy, COS, gonadotropins, oocyte maturation

## Abstract

Different Follicle Stimulating Hormone (FSH) formulation and Luteinizing Hormone (LH) are used in Assisted Reproductive Technology (ART) to induce follicles development and oocytes maturation, but it is still under debate which protocol is to be preferred. In the present study, the different effects on cumulus cells (CCs) of three controlled ovarian stimulation (COS) protocols, based on urinary FSH, recombinant FSH, or human Menopausal Gonadotropin (hMG) administration, were assessed. CCs were obtained from 42 normal-responders women undergoing COS, randomly divided into three groups according to the used gonadotropin formulation. Differences were found in the expression of genes belonging to the endocannabinoid system (the receptors *CNR1*, *CNR2* and *TRPV1*, and the enzymes involved in the metabolisms of anandamide, *NAPE-PLD* and *FAAH*, and 2-acylglycerol, *DAGL* and *MAGL*); consistently, changes in lipid (*PPAR*α, and *FASN*) and carbohydrate (*GLUT1* and *GLUT9*) metabolisms, in CCs’ macromolecules composition (highlighted by Fourier Transform Infrared Microspectroscopy, FTIRM), and in the number of retrieved oocytes were found. For the first time, statistically significant evidence on the differences related to each COS protocol on the endocannabinoid system, metabolism and macromolecular composition of CCs was found, representing a proof of concept to be further confirmed in a larger cohort of patients.

## 1. Introduction

According to the European Society of Human Reproduction and Embryology (ESHRE), Assisted Reproductive Technology (ART) allowed the conception and birth of more than 7 million babies since the first in vitro fertilization (IVF) baby was born in 1978 [1]. However, ART success rates are still unsatisfactory: in 2013, mean European pregnancy rates per aspiration were 29.6% for IVF and 27.8% per ICSI (intra-cytoplasmic sperm injection) [2]. The reasons for these low percentages are ascribable to several factors, such as male and female infertility aetiology [3,4,5], parental reproductive aging [6,7], exposure to xenobiotics [8,9], and the choice of ART treatments [10]. In particular, Controlled Ovarian Stimulation (COS) aims to induce the development of multiple dominant follicles and to mature multiple oocytes to improve probabilities of conception [11]. Among the several ovarian stimulation regimens, gonadotropin preparations represent the main formulation for COS protocols in IVF [11]. Ovarian stimulation typically involves the administration of human FSH derived from either urinary sources or recombinant techniques and/or human menopausal gonadotropin (hMG), which is derived from postmenopausal urine and has both LH and FSH activities. Since the number of high-quality oocytes to be fertilized is one of the key factors for the success of IVF, several efforts have been made to compare the effects of the different available drugs on IVF outcomes [11].

In this study, we aimed to compare the effects of urinary FSH (uFSH), recombinant FSH (rFSH) and hMG formulations: although in the literature there are several studies comparing these different stimulation protocols, no unanimous results have been reported about the best one [12,13,14,15,16,17]. Hence, in this study, we aimed to couple the traditional markers of IVF success to data obtained by biomolecular and spectral analyses to evaluate the efficacy of different treatments. The principal target of the present study was the endocannabinoid system (ECS).

The ECS is mainly composed of endocannabinoids, derivatives of fatty acids, which act as ligands of the G-protein coupled with Cannabinoid Receptors CNR1 and CNR2 [18,19,20]. Other receptors have been identified, besides CNR1 and CNR2: one of these is transient receptor potential vanilloid 1 (TRPV1) [21]. The most important endocannabinoids are anandamide (AEA) and 2-arachidonoylglycerol (2-AG), widely studied and characterized [22]. The enzymatic machinery for the modulation of AEA concentration mainly includes N–acylphosphatidylethanolamine phospholipase D (NAPE–PLD), for its synthesis [23], and the fatty acid amide hydrolase (FAAH), for its degradation [24]; regarding the concentrations of 2-AG, the diacylglycerol lipase (DAGL) regulates its biosynthesis [25] and the monoacylglyceride lipase (MAGL) regulates its degradation [26]. It is known that the endocannabinoid system plays several roles in both male and female reproduction [27,28,29]. In particular, several studies revealed that CNR1 and CNR2 receptors are expressed, in a manner that is dependent on the follicle maturation stage, by oocytes, granulosa cells of primordial, primary, secondary, tertiary follicles, corpus luteum and corpus albicans [28,30]; the same researchers also investigated the enzymatic machinery for the regulation of AEA tone, highlighting that NAPE-PLD was expressed in granulosa and theca cells of secondary and tertiary follicles and in corpus luteum and corpus albicans, while *FAAH* was present in the same follicular developmental stages, but only in theca cells [30]. A more recent study analysed the dynamic expression of *CNR1*, *CNR2*, *FAAH* and *MAGL* in granulosa cells retrieved from oocytes at GV, MI and MII stages, suggesting a role of the endocannabinoid system in regulating the nuclear maturation of the oocyte [31]. In addition, studies focusing on AEA plasma concentrations during the menstrual cycle evidenced that the highest AEA levels corresponded to ovulation, while the lowest level corresponded to the late luteal phase [32]. Endocannabinoids also target the peroxisome proliferator-activated receptors (PPARs) [33]; in particular, through the activation of PPARα or PPARγ [34], AEA and 2-AG can modulate lipid and glucose metabolism. This aspect is crucial in cumulus cells, since one of their key functions in the follicle environment is to sustain the metabolic needs of the oocytes, which are not independent [35,36,37]. Granulosa and cumulus cells have a role in follicular lipid metabolism regulation, by oxidizing lipids and storing them in lipid droplets [38].

Fourier Transform InfraRed Microspectroscopy (FTIRM) is a powerful vibrational technique, widely applied to study the biomolecular building and composition of cells and tissues, in a label-free way and providing a unique molecular fingerprint of the most important biological molecules [39,40]. FTIRM can provide, on the same sample and at the same time, an overview of the chemical composition and structure of the cellular components, by the analysis of IR bands in terms of position, intensity and width, enabling the molecular fingerprints of the most relevant biological molecules (proteins, lipids, sugars and nucleic acids) within the investigated samples. Furthermore, the obtained spectral profile can be related to biological processes, including metabolism, stress status and apoptosis [41,42]. Recently, we used FTIRM to characterize both human oocytes [43] and granulosa cells [44,45]; these cells, in particular, showed a typical spectral profile, which is dependent on several characteristics of patients, and was also influenced by the fate of the corresponding oocyte, highlighting the feasibility and the potential to study these cells with FTIRM to obtain information on the ovarian functionalities in general and on oocyte status.

Based on the role of the endocannabinoid system on the oocyte maturation process and the importance of CCs’ metabolism for the quality of associated oocyte, we aimed to couple biomolecular and vibrational spectroscopy analyses to provide new information on the mechanisms of action of different ovarian stimulation protocols on the endocannabinoid system and lipid and carbohydrate metabolism of the CCs and the number of total retrieved oocytes.

## 2. Results

### 2.1. Patients’ Data

Table 1 reports patients’ data (including age, Body Mass Index, FSH and Anti Müllerian Hormone serum levels), together with the number of total retrieved oocytes and the percentages of immature and mature oocytes. Data did not highlight statistically significant differences related to age, BMI, FSH and AMH serum levels; conversely, significant differences were found in the number of retrieved oocytes, which was significantly higher in the uFSH experimental group, with respect to rFSH and hMG (statistically comparable). No significant difference was reported among the three experimental groups, related to the percentage of immature and mature retrieved oocytes.

### 2.2. Gene Expression Analysis

CCs samples of women treated with uFSH, or rFSH, or hMG stimulation protocols were first analysed by PCR for gene expression analysis, in order to assess the different effects of these treatments on the modulation of the endocannabinoid system and metabolism with respect to the administered gonadotropins. The primer sequences used are reported in Table 2 (see Materials and Methods section).

**Figure 1:**
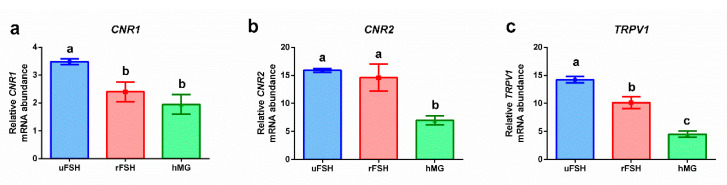
Gene expression profiles: (**a**) *CNR1*: cannabinoid receptor 1; (**b**) *CNR2*: cannabinoid receptor 2; (**c**) *TRPV1*: transient receptor potential cation channel subfamily V member 1. Results are reported as mean ± standard deviation. Statistical significance was set at *p* < 0.05 and calculated by ANOVA followed by Tukey’s multiple comparisons test. Different letters above columns indicate statistical differences among groups.

Figure 1 reports endocannabinoid receptors’ modulation brought about by different COS treatments. A significantly higher level of gene expression was observed in CCs from patients stimulated with uFSH; the lowest level of gene expression was reported for the hMG experimental group. The rFSH experimental group was characterized by expression levels comparable to those of the hMG group (*CNR1*), comparable to those of the uFSH group (*CNR2*), or intermediate between the uFSH and hMG experimental groups.

**Figure 2:**
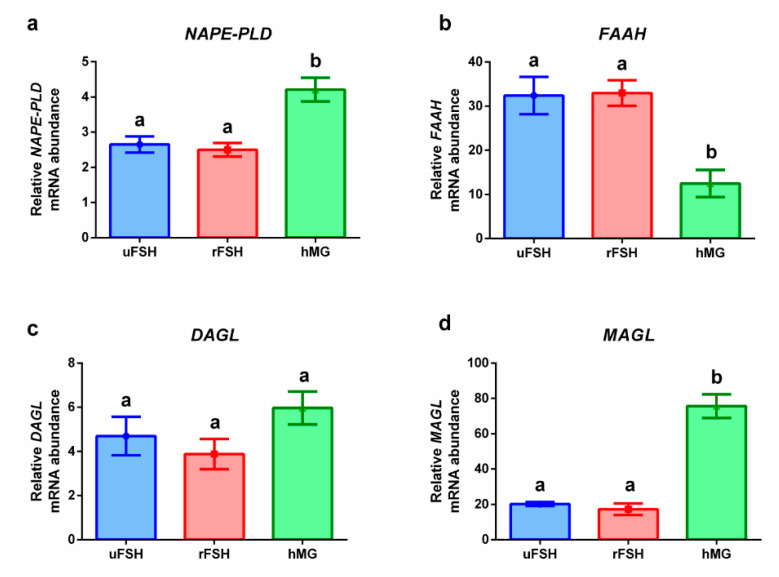
Gene expression profiles: (**a**) *NAPE-PLD*: N-acyl phosphatidylethanolamine phospholipase D; (**b**) *FAAH*: fatty acid amide hydrolase; (**c**) *DAGL*: diacylglycerol lipase alpha/beta; (**d**) *MAGL*: monoglyceride lypase. Results are reported as mean ± standard deviation. Statistical significance was set at *p* < 0.05 and calculated by One Way ANOVA followed by Tukey’s multiple comparisons test. Different letters above columns indicate statistical differences among groups.

Figure 2 shows the expression levels of genes coding for the enzymatic machinery involved in the synthesis and degradation of the two endocannabinoids, the AEA (Figure 2a,b) and the 2-AG (Figure 2c,d). Significantly higher levels (*p* < 0.05) of gene expression were evidenced for *NAPE-PLD*, responsible of AEA synthesis, in the hMG experimental group, concomitantly with a significant lower (*p* < 0.05) value of gene expression of *FAAH*, responsible for the AEA degradation; the expression levels of the uFSH and rFSH experimental groups were comparable (Figure 2a,b). Conversely, the genes coding for the enzymes involved in the modulation of 2-AG concentration displayed a different trend: *DAGL* expression levels were statistically comparable among experimental groups, while the expression of *MAGL*, responsible for the degradation of 2-AG, was significantly higher in the hMG experimental group with respect to both uFSH and rFSH treatments, which showed similar levels (Figure 2c,d).

**Figure 3:**
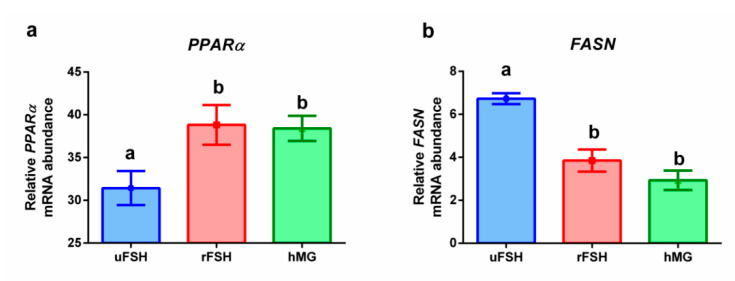
Gene expression profiles: (**a**) *PPARα*: peroxisome proliferator activated receptor alpha; (**b**) *FASN*: fatty acid synthase. Results are reported as mean ± standard deviation. Statistical significance was set at *p* < 0.05 and calculated by ANOVA followed by Tukey’s multiple comparisons test. Different letters above columns indicate statistical differences among groups.

Figure 3 reports the results of PCR analysis for genes involved in lipid metabolism of CCs. A statistically significantly higher expression level of peroxisome proliferator-activated receptors, *PPARα*, is reported for rFSH and hMG groups, with respect to uFSH (Figure 3a). Conversely, the expression levels of the enzyme fatty acid synthase (*FASN*) were significantly lower in rFSH and hMG experimental groups, with respect to uFSH (Figure 3b).

**Figure 4:**
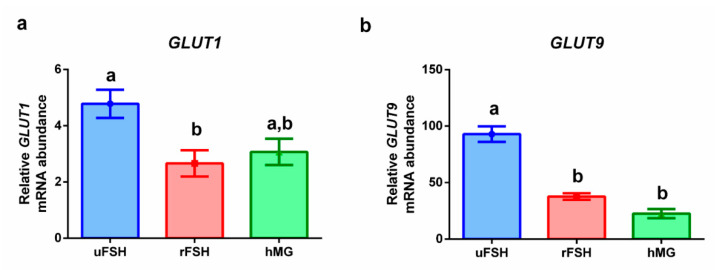
Gene expression profiles: (**a**) *GLUT1*: glucose transporter 1 (*SLC2A1*: solute carrier family 2 member 1); (**b**) *GLUT9*: glucose transporter 9 (*SLC2A9*: solute carrier family 2 member 9). Results are reported as mean ± standard deviation. Statistical significance was set at *p* < 0.05 and calculated by ANOVA followed by Tukey’s multiple comparisons test. Different letters above columns indicate statistical differences among groups.

Figure 4 shows the expression levels of genes involved in glucose transport. Statistically significantly lower expression levels were found in both the rFSH and hMG experimental groups for both of the investigated glucose transporters, *GLUT1* (Figure 4a) and *GLUT9* (Figure 4b).

### 2.3. FTIRM Analysis

CCs samples of women treated with uFSH, rFSH and hMG stimulation protocols were then analysed by FTIRM, in order to assess the different effects of these treatments on their biochemical profile.

**Figure 5:**
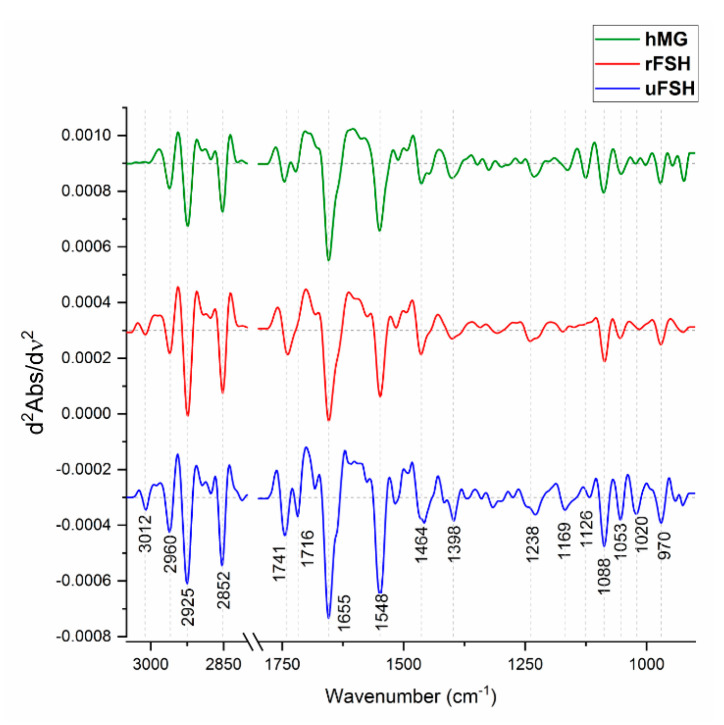
Analysis of spectral profiles. Average spectra of uFSH, rFSH and hMG experimental groups, reported in second derivative mode (3050–900 cm^−1^). The wavenumbers of the most relevant peaks are shown at the bottom.

Figure 5 displays second derivative of average spectra of the three examined experimental groups. Second derivative spectra were used to precisely identify the spectral features characterizing rFSH-, uFSH- and hMG-treated CCs. The most significant peaks are reported in Table 3 (see Materials and Methods section), in terms of wavenumbers, vibrational modes and biochemical assignments.

**Figure 6:**
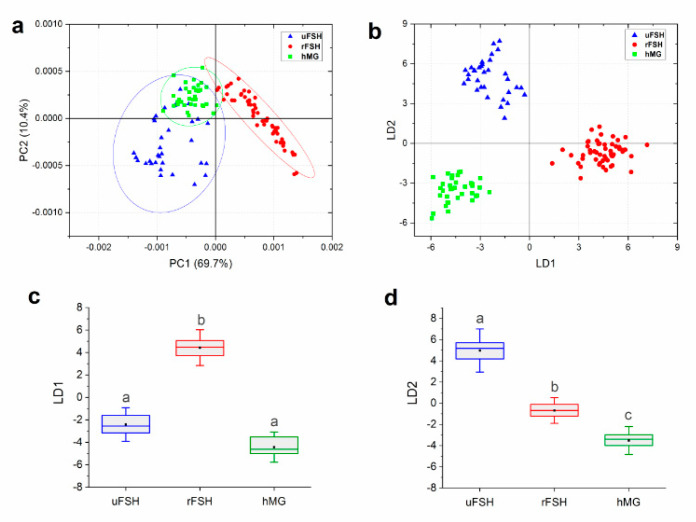
Multivariate statistical analysis of spectral data: (**a**) scores plot of the 2 first principal components after PCA performed on IR spectra collected from uFSH, rFSH and hMG CCs samples. The percentage of variance explained by each Principal Component (PC) is indicated inside parentheses. Ellipses indicate 95% confidence. (**b**) Principal Component Analysis-Linear Discriminant Analysis (PCA–LDA) scores plot for uFSH, rFSH and hMG CCs samples. LD1 and LD2 represent the first and second linear discriminant functions obtained by the canonical variables scores of PCA–LDA. (**c**) One-dimensional scores plots of LD1 and (**d**) LD2 for uFSH, rFSH and hMG CCs samples. Box chart legend: center line = median; black square = mean; edges = 25th and 75th percentile; whiskers = standard deviation. Different letters over box charts indicate statistically significant differences among groups (one-way ANOVA and Tukey’s multiple comparisons test). Statistical significance was set at *p* < 0.05.

Average absorbance spectra and their corresponding ± standard deviation spectra were calculated for each patient and submitted to multivariate statistical analyses. Principal Component Analysis coupled with Linear Discriminant Analysis (PCA–LDA) was performed to highlight the spectral features discriminating CCs treated with the different ovarian stimulation protocols (Figure 6). First, PCA was performed, and PC scores (13 PCs: 95.17% cumulative explained variance) were used as input variables for LDA. The PCA scores plot highlighted a clear segregation along the PC1 (69.7% of explained variance) of rFSH group with respect to the uFSH and hMG groups, while a slight superimposition along PC1 was observed for the uFSH and hMG groups, which were separated along PC2 (10.4% of explained variance) (Figure 6a). The coupling of PCA and LDA improved and clarified the discrimination of the three experimental groups (Figure 6b). Figure 6c,d report the one-dimensional scores plots of LD1 and LD2, respectively, and confirm the importance of LD1 in discriminating the rFSH group from the uFSH and hMG groups, and of LD2 in discriminating each experimental group from the others.

**Figure 7:**
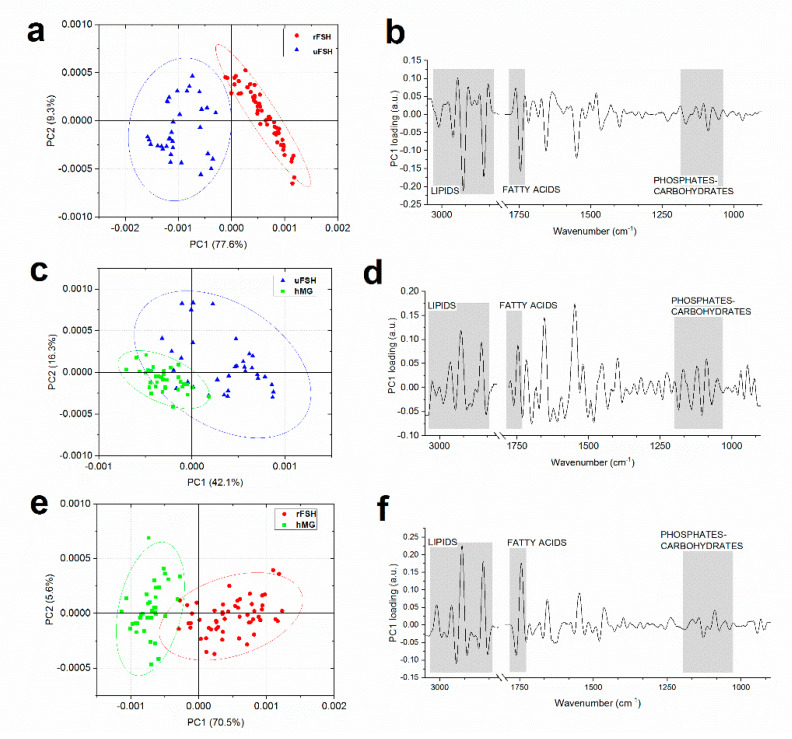
Pairwise PCA of spectral data. PCA scores plot of (**a**) “uFSH vs. rFSH”, (**c**) “uFSH vs. hMG”, and (**e**) “rFSH vs. hMG” (uFSH, blue triangles; rFSH, green squares; hMG, red circles). The percentage of variance explained by each PC is indicated inside parenthesis. Ellipses indicate 95% confidence. PC1 loadings of (**b**) “uFSH vs. rFSH”; (**d**) “uFSH vs. hMG” and (**f**) “rFSH vs. hMG” pair-wise comparisons. Grey rectangles indicate the most discriminant spectral ranges.

For a deeper understanding of the spectral features differentiating the three experimental groups, pairwise PCA was performed. Figure 7 displays the pair-wise scores plots and the corresponding PC1 loadings. In the “uFSH vs. rFSH” scores plot, a clear discrimination along PC1 (77.6% of explained variance) between CCs of the two experimental groups was highlighted (Figure 7a); also the “rFSH vs. hMG” scores plot showed a good, but not complete, segregation of observations along PC1 (70.5% of explained variance) (Figure 7c); on the contrary, the “uFSH vs. hMG” scores plot did not highlight a good discrimination between the two experimental groups, the observations of which partially overlap, even if the separation mostly occurs along PC1 (42.1% of explained variance) (Figure 7e). The comparison of PC1 loadings of “uFSH vs. rFSH”, “rFSH vs. hMG” and “uFSH vs. hMG” (Figure 7b,d,f) comparisons revealed that spectral modifications mainly appear in the following regions: 3050–2800 cm^−1^ (characteristic of lipids), 1760–1700 cm^−1^ (characteristic of fatty acids), and 1170–1050 cm^−1^ (characteristic of phosphates and carbohydrates).

**Figure 8:**
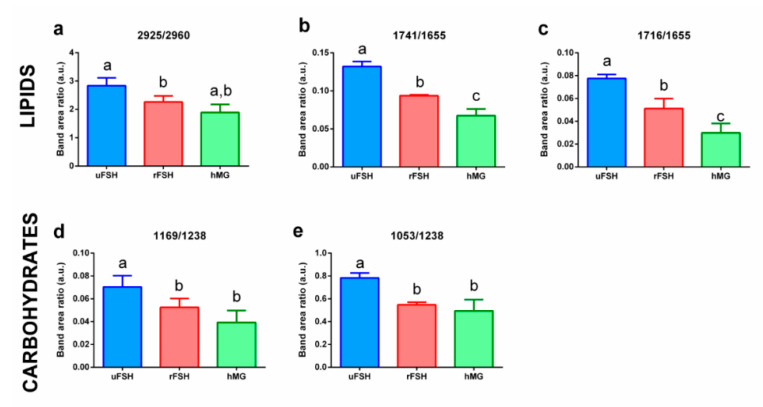
Results of ANOVA test on band area ratios 2925/2960 (**a**), 1741/1655 (**b**), 1716/1655 (**c**), 1169/1238 (**d**), 1053/1238 (**e**). Histograms show the values of the 5 most discriminant band area ratios obtained by peak fitting procedure on mean spectra and mean ± standard deviation spectra of the uFSH, rFSH and hMG experimental groups. Data are reported as mean ± standard deviation. Different letters above histograms indicate a statistically significant difference among groups. Statistical significance was set at *p* < 0.05 and calculated by ANOVA followed by Tukey’s multiple comparisons test.

To quantify the modifications affecting the above defined spectral regions, average absorbance spectra of uFSH, rFSH and hMG CCs samples and their average ± standard deviation spectra were curve fitted. Specific band area ratios were calculated and analysed; Figure 8 displays the most discriminant spectral differences among the three experimental groups. The other calculated band area ratios, representative of the relative amount of lipids’ and proteins’ methyl and methylene groups (1464/1655 and 1398/1655), and of the relative amount of phosphates (1116/1238 and 1088/1238) did not change significantly (*p* > 0.05) among the experimental groups. Both lipids’ and carbohydrates’ metabolism of CCs appeared affected by the different ovarian stimulation protocols: lipid component is affected in terms of length of lipid alkyl chains (2925/2960, Figure 8a), triglycerides amount (1741/1655, Figure 8b) and fatty acids amount (1716/1655, Figure 8c), which are significantly higher in the uFSH experimental group, with respect to both of the others. In addition, the carbohydrate metabolism was different based on the selected stimulation protocol, with a higher content of carbohydrates (1169/1238 and 1053/1238, Figure 8d,e) in the uFSH experimental group and lower ones in rFSH and hMG groups.

## 3. Discussion

In ART procedures, controlled ovarian stimulation (COS) is used to induce the development of multiple dominant follicles and to mature multiple oocytes [11]. Besides the variety of available molecules, gonadotropins (FSH and LH) represent the main formulation for COS protocols [46]. Since the number of high-quality oocytes to be fertilized is one of the key factors for the success of IVF, several efforts have been made to compare the effects of the different available drugs on IVF outcomes [11]. Nevertheless, there is still no consensus among researchers and clinicians about the optimal protocol to choose and the best effective combination of gonadotropins to use. According to previous studies, rFSH is more effective than uFSH in terms of number of retrieved oocytes, number of obtained embryos [12] and pregnancy rates [13]. Other studies revealed significantly higher fertilization rates and a higher number of good quality embryos when using uFSH with respect to rFSH [14,15]; while other data showed no significantly detectable differences in terms of number and quality of oocytes and pregnancy rates between rFSH and uFSH formulations [14,16]. Similarly, no significant differences were reported in terms of fertilization and pregnancy rates between uFSH and hMG [17].

In this study, we aimed to compare the effects of uFSH, rFSH and hMG formulations on diverse biomolecular aspects of cumulus cells, given their crucial role in follicular development and oocyte maturation [44,47,48,49]. In particular, we focused on the expression of genes involved in the endocannabinoid system in addition to some genes involved in lipid and carbohydrate metabolisms in CCs. Changes in the macromolecular profile of CCs related to gonadotropin formulations were monitored by FTIRM.

It is known that the endocannabinoid system plays several roles in both male and female reproduction [27,28,29] and several recent studies revealed a dynamic expression of some elements of the endocannabinoid system, related to the follicle and oocyte maturation stage [28,30,31].

In our study, cumulus cells were all retrieved, as in IVF standard procedures, with ovulation triggering performed when at least N.3 follicles had a diameter of >18 mm, avoiding biases related to the dependency of endocannabinoids to the follicle developmental stage. Our results revealed a modulation of the expression of the genes encoding for CNR1, CNR2 and TRPV1 receptors according to the gonadotropin preparations used for the ovarian stimulation of patients. In particular, the uFSH group was characterized by the highest levels of expression of the three genes, the rFSH group was characterized by intermediate values, while the hMG group showed the lowest expression values. These results suggest that, even if structurally similar, purified and recombinant FSHs determine a different activation of the endocannabinoid system; moreover, the combination of the purified FSH with purified LH (hMG) results in a significantly lower expression of genes related to endocannabinoid receptors. CNR1 and CNR2 were found to be expressed both in oocyte and follicular cells in pre-ovulatory follicles [30], thus suggesting an involvement of the endocannabinoid system in both the nuclear maturation of the oocyte and the steroidogenic activity of the follicle. CNR1 and CNR2 are seven-transmembrane-spanning G-protein-coupled receptors, and the binding of AEA or 2-AG triggers diverse signal transduction mechanisms, including the inhibition of adenylate cyclase, with a consequent decrease in cAMP levels, and the inhibition of some calcium channels, with a reduction in calcium influx; hence, these cascades regulate mechanisms of growth, proliferation and differentiation [50]. Since the use of different gonadotropin formulations has been shown to modulate the expression of the genes encoding for CNR1, CNR2, and TRPV1 receptors, we can hypothesize a strong influence of the chosen COS treatment on crucial ovarian processes, including oocyte’s resumption of meiosis and nuclear maturation, and follicle growth.

Given the known importance of AEA in ovarian functions, the enzymes involved in its synthesis and degradation, NAPE-PLD and FAAH, were investigated: the hMG group was characterized by the highest expression of *NAPE-PLD* and the lowest expression of *FAAH*, suggesting a concentration of AEA in these cumulus cells higher than the one hypothesized in the uFSH and rFSH groups. El-Talatini and colleagues reported that NAPE-PLD was not detectable in the oocyte at the stage of tertiary follicle [30], allowing the hypothesis that AEA is transported from ovarian cells to the oocyte, which does not synthesize it by itself. Hence, our results about the expression of the *NAPE-PLD* gene, besides not giving information about the protein status, may also suggest a higher concentration of AEA in the oocytes of women stimulated with hMG preparations. Regarding the enzymatic machinery of synthesis and degradation of AEA, the uFSH and rFSH experimental groups did not show significant differences, which are, hence, ascribable only to the addition of LH to the drug formulation.

Less is known about the role in ovarian functions of 2-AG, the other most studied endocannabinoid. The expression of *DAGL*, responsible for the synthesis of 2-AG, did not show significant modifications among experimental groups, while *MAGL* was significantly more expressed in the hMG experimental group. This result suggests that different gonadotropin formulations do not impact on the synthesis of 2-AG, but deeply alter its degradation, in particular when adding LH to the stimulation protocol.

The clinical results related to the number of retrieved oocytes here found suggested that the combination of purified FSH and LH was the least efficient protocol in terms of the number of retrieved oocytes. In the literature, a relation between AEA and LH is reported: endogenous AEA inhibits the release of LH [51,52]. Since CCs from the hMG group appeared to produce more AEA than the others, we may suggest that the block of the LH secretion—due to the treatment with GnRH antagonist—and the subsequent administration of this gonadotropin may determine a still unknown mechanism of positive feedback, which increases AEA production. Coupling the clinical results with those related to the endocannabinoid system’s regulation in CCs, it is evident that further studies are necessary to prove the “negative” influence of AEA on follicle maturation and oocyte quality, and the possible “positive” role of 2-AG.

Endocannabinoids AEA and 2-AG are known to modulate lipid and glucose metabolism through the activation of PPARα or PPARγ [34]. Given the role of cumulus cells in sustaining oocyte with essential nutrients, this aspect of the research is crucial. PPARα is involved in the regulation of fatty acid catabolism and is known to be activated by some endocannabinoids, including AEA [34]. We investigated the expression of genes encoding for PPARα and FASN, the fatty acid synthase: we found that the expression of *FASN* was significantly reduced in rFSH and even more in hMG experimental groups with respect to uFSH; consistently, *PPARα*, responsible for the catabolism of fatty acids, showed an opposite trend, with the highest expression levels in the hMG group. These results were supported by FTIRM analysis: pairwise PCAs highlighted that the two most relevant spectral features discriminating the experimental groups were in the 3050–2800 cm^−1^ spectral region, characteristic of lipids in general, and the 1760–1700 cm^−1^ spectral region, characteristic of fatty acids. The peak fitting analysis confirmed that the decreased expression of *FASN* and the increased expression of *PPARα* were consistently related to reduced length of lipid alkyl chains and lower content of triglycerides and fatty acids. These results are supported by the known importance of cumulus cell lipid metabolism in maintaining metabolic homeostasis of the follicle: a decrease in fatty acid synthase activity may impair the optimal environment in which oocytes are enclosed. Our previous results on granulosa cells by FTIRM evidenced a similar result: GCs of healthy women were characterized by higher levels of the CH_2_/CH_3_ ratio, with respect to granulosa cells retrieved from women affected by ovarian endometriosis [45].

The glucose metabolism can also be modulated by endocannabinoids. Cumulus cells support oocytes in the uptake and utilization of glucose, via aerobic glycolysis [38]. Glucose is fundamental for oocytes, but it needs to be previously converted into pyruvate by granulosa cells [53]. Granulosa and cumulus cells take up glucose via sodium-coupled glucose transporters (SGLTs) or through facilitative glucose transporters (GLUTs) [54]. In the present study, *GLUT1* and *GLUT9* have been selected among the other GLUTs, because they displayed a different expression pattern in granulosa cells [55]. In particular, some GLUTs, like *GLUT1*, are constitutively expressed in granulosa cells, while others, including *GLUT9*, are differentially expressed according to metabolic status. Moreover, it is reported in the literature that the expression of GLUT9 statistically correlates with the rates of in vitro maturation of oocytes [55]. Our PCR results showed lower levels of *GLUT1* and *GLUT9* expression in both rFSH and hMG groups, with respect to uFSH, which displayed the highest expression levels. These data were confirmed by FTIRM analysis results: for both rFSH and hMG CCs, the peak fitting procedure revealed a decrease in the 1169/1238 and 1053/1238 band area ratios, assigned to carbohydrates. A decrease in glucose transport and content in cumulus cells may be a signal of a less efficient glucose metabolism activity in these cells [45,53], which could affect the oocytes’ recruitment, as suggested by the lower number of retrieved oocytes in the rFSH and hMG experimental groups.

## 4. Materials and Methods

### 4.1. Ethical Approval

The study, approved by the ethics committee N. 2020241, was carried out in full accordance with ethical principles for experiments involving humans, including The Code of Ethics of the World Medical Association (Declaration of Helsinki, 2013). Patients participating in the investigation signed an informed consent agreement, which included the donation of CCs. All samples were strictly anonymous, making it impossible to correlate them to patients.

### 4.2. Patient Population and Experimental Design

A cohort of N. 42 patients were enrolled in an IVF program, according to specific inclusion criteria: age between 32 and 40 years; non-smokers; BMI 20–26 kg/m^2^; primary infertility; AMH level between 1 and 2.5 ng/mL; 2nd day FSH < 12 mU/mL. Exclusion criteria followed for the cohort selection were pelvic endometriosis, previous ovarian surgery, presence of any ovarian mass, pelvic inflammatory disease, autoimmune or chronic systemic diseases.

Normal responder women were grouped according to the gonadotropin formulation used for ovarian stimulation: (i) N. 10 patients stimulated with uFSH (Fostimon^®^, Ibsa Farmaceutici, Lodi, Italia); (ii) N. 18 patients stimulated with rFSH formulations (Puregon^®^, MSD, Roma, Italia; Gonal-F^®^, Merck, Darmstadt, Germany; Ovaleap^®^, London, Theramex, UK); (iii) N. 14 patients stimulated with hMG formulations (Meropur^®^, Ferring, Sweden). All groups were matched by inclusion criteria.

In all cases, short protocol of gonadotropin COS was used (daily subcutaneous injection started on the 2nd day of menstrual cycle), GnRH antagonist (Cetrotide, Merck, Darmstadt, Germany; Orgalutran, MSD, Roma, Italia) was administered on initiation by a follicle of 12–14mm, and HCG 10.000 UI was used for ovulation triggering 36 h before oocyte retrieval. The gonadotropin doses for patients included in the study were statistically comparable. An ultrasound transvaginal scan (DC-70, Mindray Medical Italy S.R.L.) for follicular growth monitoring was performed during COS.

### 4.3. CCs Samples Collection and Preparation

Oocyte collection was carried out under echographic control, with the use of a needle (Ovum aspiration needle 37ga–35 cm, COOK Medical, Bloomington, IN, USA) filled with buffered medium (Flushing Medium with Heparin 10 IU/mL) and applied to a transvaginal probe, in order to aspirate follicular fluid and retrieve oocytes. Follicular fluid was recovered in a test tube, which was maintained at 37 °C, and then poured in a Petri dish (Thermo Fisher Scientific Inc., Waltham, MA, USA) for visualization by a stereo microscope (Stemi 508, provided with a thermo plate; ZEISS, Oberkochen, Germany), in order to identify the cumulus and oocyte cells’ complexes (COCs). COCs were washed with a buffered medium (Flushing Medium, Origio, CooperSurgical, Denmark) and subsequently transferred into a test tube within the same buffered and pre-equilibrated culture medium at 37 °C.

In order to remove CCs and denudate oocytes, one of the four wells of the IVF-4-Well Dish (Thermo Fisher Scientific Inc., USA) was filled with 150 μL of pre-equilibrated buffered culture medium (Flushing Medium, Origio, CooperSurgical, Målov, Denmark) and 150 μL of pre-warmed (37 °C) SynVitro Hyadase (Origio, CooperSurgical, Målov, Denmark); the other three wells were filled with only pre-equilibrated buffered culture medium. COCs were placed in the well with SynVitro Hyadase and gently aspirated up and down by a denuding pipette (Flexipet^®^ denuding pipette, COOK Medical, Bloomington, IN, USA). Oocytes were transferred into a well rinsed with culture medium and the remaining cumulus cells were removed. Oocytes were assessed for nuclear maturity: oocytes with extruded first polar body were considered mature (metaphase II, MII); both oocytes at the germinal vesicle (GV) stage and metaphase I (MI) stage were generally taken into account as “immature”.

In order to remove the culture medium and prepare cells for further analyses, CCs samples were centrifuged at 1000× *g* for 15 min and washed using physiologic salt solution (Merck, Germany); the procedure was repeated twice. The final CCs pellet was resuspended in 500 μL of a physiologic salt solution, and divided according to the subsequent analyses: 10 μL of the cell suspension were fixed in a 4% paraformaldehyde (PFA) solution for 10 min, washed twice in physiological salt solution, and then stored at 4 °C until FTIR measurements; the remaining cells were centrifuged, and the pellet was stored at −80 °C until gene expression analysis.

### 4.4. RNA Extraction and cDNA Synthesis

Total RNA extraction from CCs was performed using the miRNeasy Mini Kit^®^ (Qiagen, Hilden, Germany) extraction kit, following the manufacturer’s protocol. Total RNA extracts were eluted in 30 μL of RNAse-free water. Final RNA concentrations were determined with a NanoDrop™ 1000 Spectrophotometer (Thermo Fisher Scientific Inc., Waltham, MA, USA) and RNA integrity was verified by Gel Red (Biotium Inc., Fremont, CA, USA) staining of 28S and 18S ribosomal RNA fragments on 1% agarose gel. RNA was stored at −80 °C until use. Total RNA was treated with DNAse (10 IU at 37 °C for 10 min, Thermo Fisher Scientific Inc., Waltham, MA, USA); a total amount of 100 ng of RNA was used for cDNA synthesis, employing the SuperScript-II cDNA Synthesis Kit (Invitrogen, Thermo Fisher Scientific Inc., Waltham, MA, USA).

### 4.5. Real-time Polymerase Chain Reaction

Relative quantification of gene expression was performed with the SYBR Green method in a CFX96 Real-Time PCR system (Bio-Rad, Hercules, CA, USA). Duplicate PCRs were carried out for each sample analysed. Reactions were set mixing 1 μL diluted (1/10) cDNA, 5 μL of 2× SYBR Green PCR Master Mix (Bio-Rad, USA), containing SYBR Green as a fluorescent intercalating agent, 0.2 μM of forward and reverse primers, and 3.8 μL of milliQ water.

The thermal profile was as follows: enzyme activation at 95 °C for 3 min; 45 cycles of denaturation (10 s at 95 °C), followed by 20-s annealing at 60 °C for all the examined genes, and 20-s elongation at 72 °C. Fluorescence was monitored at the end of each cycle. Dissociation curves for primer specificity and absence of primer–dimer formation check were performed and consistently showed a single peak. Ribosomal protein L13a (*RPL13A*) and β-actin (*ACTB*) were used as internal controls to enable result standardization by eliminating variations in mRNA and cDNA quantity and quality [56]. These genes were chosen because their mRNA levels did not vary between experimental groups. No amplification products were observed in negative controls and no primer–dimer formations were observed in the control templates. Data were analysed using CFX Manager Software version 3.1 (Bio-Rad, USA). The quantification method was based on a ∆∆Ct calculation implemented with the Pfaffl equation, to improve accuracy by accounting for varied reaction efficiencies depending on primers [57,58]. Modification of gene expression is represented with respect to zero. The primer sequences used are reported in Table 2.

### 4.6. FTIRM Analysis

FTIRM measurements were performed at the Chemical and Life Sciences branch of the infrared Beamline SISSI (Synchrotron Infrared Source for Spectroscopic and Imaging), Elettra Sincrotrone Trieste (Trieste, Italy) (Proposal N. 20180140). A Hyperion 3000 Vis-IR microscope equipped with a HgCdTe (MCT_A) detector and coupled with a Vertex 70V interferometer (Bruker Optics, Ettlingen, Germany) was used.

CCs from each patient were divided into three aliquots separately analysed by FTIRM. Ten μL-aliquots of each CCs sample were deposited after the PFA fixation process onto CaF_2_ optical windows (1-mm thick, 13-mm diameter) and placed into a specific in-house built biocompatible IR transparent microfluidic device for FTIRM analysis of in vitro samples. The device consisted of two CaF_2_ optical windows (0.5 mm thick, 13 and 10 mm diameter respectively), spaced 7.5 μm apart [59]. From each aliquot of CCs sample, ~10 microareas (30 × 30 μm^2^) containing densely packed cell monolayers were selected by visible microscopy, on which IR spectra were collected in transmission mode in the Medium InfraRed (MIR) region (4000–800 cm^−1^) [44], averaging 512 scans, with a spectral resolution of 4 cm^−1^, zero-filling factor of 2, and a scanner velocity of 40 kHz. Background spectra were collected using the same parameters on clean zones of the CaF_2_ optical windows. All raw IR spectra underwent the following pre-processing procedure. The spectral contributions of water were subtracted by using an in-house optimized routine [59]; then, subtracted spectra were also corrected for the contribution of atmospheric carbon dioxide and water vapour (Atmospheric compensation routine, OPUS 7.1 software, Bruker Optics, Ettlingen, Germany) and vector normalized in the 4000–800 cm^−1^ range (Normalization routine, OPUS 7.1 software). Then, spectra were evaluated based on the height of the band centred at ~1660 cm^−1^ (Amide I band of proteins), which is always the highest peak of cell spectra; average spectra having at 1660 cm^−1^ an absorbance value lower than 0.07 a.u. (~20%) were discarded [41]. On these preprocessed spectra, for each patient, the average absorbance spectrum and its corresponding ± standard deviation spectra were calculated and submitted, after conversion in second derivative mode (Savitzky-Golay filter, 13 points of smoothing) to Principal Component Analysis (PCA), by using the “Principal Component Analysis for Spectroscopy” tool on OriginPro 2018b (OriginLab Corporation, Northampton, MA, USA). PCA was performed both with all the experimental groups and in a pair-wise manner (uFSH vs. rFSH, uFSH vs. hMG, rFSH vs. hMG). To highlight the spectral variations among the experimental groups, PC loadings were analysed. Moreover, for the analysis with all the experimental groups, PCA was coupled with Linear Discriminant Analysis (LDA): in spectroscopy, PCA is often used to reduce the spectral dataset to PC scores; then, LDA is performed using PC scores as variables [60,61] (OriginPro 2018b). The number of PCs selected for LDA represented 95% of the cumulative explained variance.

Average absorbance spectra and their corresponding ± standard deviation spectra were curve fitted in the following regions: 3050–2800 cm^−1^ (representative of lipids), 1770–1350 cm^−1^ (representative of fatty acids and proteins), and 1350–900 cm^−1^ (representative of phosphate groups and carbohydrates). The selected spectral regions were fitted with Gaussian band components upon straight baseline correction and vector normalization (GRAMS/AI 7.02, Galactic Industries, Inc., Salem, NH, USA). The underlying bands were precisely identified by second derivative results, and their centre values (expressed as wavenumbers) together with the corresponding vibrational mode and biochemical assignment are listed in Table 3. For each fitted region, the integrated areas of selected underlying bands were calculated and used to obtain the following band area ratios: 2925/2960 (length of lipid alkyl chains); 1741/1655 (relative amount of triglycerides), 1716/1655 (relative amount of fatty acids); 1464/1655 and 1398/1655 (relative amount of lipids and proteins methyl and methylene groups); 1169/1238 and 1053/1238 (relative amount of carbohydrates); 1116/1238 and 1088/1238 (relative amount of phosphates).

### 4.7. Statistical Analysis

Band area ratios and gene expression analysis results are reported as mean ± standard deviation. Significant differences between experimental groups were determined by means of factorial analysis of variance (one-way ANOVA), followed by Tukey’s multiple comparisons test. The analysis of mature and immature oocytes was performed by Chi Square test (Prism6; Graphpad Software, Inc., San Diego, CA, USA). Significance was set at *p* < 0.05. The statistical software OriginPro 2018b was used.

## 5. Conclusions

To conclude, the multidisciplinary approach here presented shed new light on different mechanisms of action and effects of different gonadotropin formulations used in ovarian stimulation protocols. Particularly, we focused on the modulation of the endocannabinoid system and on lipid and glucose metabolism, coupled with vibrational information related to the biochemical profile of cumulus cells. Based on our results, we suggest that:

- Even if structurally similar, purified and recombinant FSH probably act differently in modulating ovarian functions, since most of the results evidenced different effects on cumulus cells.
- The genes codifying for the endocannabinoid receptors CNR1, CNR2, and TRPV1 are modulated by the type of gonadotropins used for the stimulation. Given their hypothesized role in the nuclear maturation of the oocyte and in the steroidogenic activity of the follicle, these results suggest that the choice of COS formulations may impact the main ovarian functions.
- The genes codifying for the enzymes involved in the endocannabinoid synthesis and degradation are modulated by the type of gonadotropins used for the stimulation. The differences found in CCs from hMG group on the expression of genes involved in AEA production and degradation made it possible to hypothesize an increase in AEA that, given the reduced number of total retrieved oocytes reported in the study, may be detrimental for IVF success rates. Less is known about the role of 2-AG on ovarian functions: the least efficient stimulation protocol (hMG) is characterized by the highest levels of *MAGL* expression, making it possible to hypothesize a reduced concentration of 2-AG. These results suggest that the modulation of the endocannabinoid system by gonadotropins can involve 2-AG and its decrease may be detrimental to IVF success rates.
- Lipid and glucose metabolism was different, depending on the gonadotropins selected for the stimulation. A decreased fatty acid synthase activity, with consequent decreased fatty acids content, may impair the optimal environment in which oocytes are enclosed, since lipid metabolism provides the energy necessary for protein synthesis during the nuclear maturation of the oocyte. Glucose is internalized by cumulus cells, for its subsequent conversion into pyruvate and oocyte’s utilization; hence, decreased glucose transport and content in cumulus cells negatively affect the oocyte’s maturation and recruitment, as confirmed by the lower number of retrieved oocytes in the rFSH and hMG groups.

## Figures and Tables

**Table 1:** Summary of patients’ data calculated within urinary FSH (uFSH), recombinant FSH (rFSH) and human Menopausal Gonadotropin (hMG) experimental groups: age; BMI (Body Mass Index), FSH (Follicle Stimulating Hormone) and AMH (Anti Müllerian Hormone) serum levels; number of total retrieved oocytes, percentage of immature and mature oocytes. Data of age, BMI FSH, AMH and number of retrieved oocytes are reported as mean ± standard deviation. Asterisks indicate a statistically significant difference among the three experimental groups. Statistical significance was set at *p* < 0.05 and calculated by ANOVA followed by Tukey’s multiple comparisons test (age, BMI, FSH, AMH and average retrieved oocytes) and by Chi square test (% immature and % mature oocytes).

Group	Age	BMI	FSH	AMH	Average Retrieved Oocytes	% Immature Oocytes	% Mature Oocytes
uFSH (N. 10)	35.60 ± 3.20	24.91 ± 1.83	6.53 ± 1.83	2.62 ± 1.64	7.90 ± 2.42	21.80%	78.20%
rFSH (N. 18)	36.22 ± 4.90	23.82 ± 2.09	6.21 ± 2.09	2.12 ± 1.03	5.61 ± 3.22 *	18.01%	81.99%
hMG (N. 14)	37.57 ± 2.95	23.88 ± 1.21	5.78 ± 1.48	1.85 ± 0.93	5.53 ± 2.99 *	21.87%	78.13%

**Table 2:** List of primers used for gene expression analyses by Real-Time qPCR (*ACTB*: actin beta; *RPL13A*: ribosomal protein L13a; *TRPV1*: transient receptor potential cation channel subfamily V member 1; *CNR1*: cannabinoid receptor 1; *CNR2*: cannabinoid receptor 2; *NAPE-PLD*: N-acyl phosphatidylethanolamine phospholipase D; *FAAH*: fatty acid amide hydrolase; *DAGL*: diacylglycerol lipase; *MAGL*: monoglyceride lypase; *PPARα*: peroxisome proliferator activated receptor alpha; *FASN*: fatty acid synthase; *GLUT1*: glucose transporter 1 (*SLC2A1*: solute carrier family 2 member 1); *GLUT9*: glucose transporter 9 (*SLC2A9*: solute carrier family 2 member 9)).

Gene	Forward Primer (5′–3′)	Reverse Primer (5′–3′)	Accession Number
*ACTB*	GCAGAAGGAGATCACATCCCTGGC	CATTGCCGTCACCTTCACCGTTC	NM_001101.5
*RPL13A*	TCTGGAGGACTGTAAGAGGTATGC	AGACGCACAATCTTGAGAGCAG	NM_012423.3
*TRPV1*	GACTTCAAGGCTGTCTTCATCATCC	CAGGGAGAAGCTCAGGGTGCGC	NM_018727.5
*CNR1*	CCACTCCCGCAGCCTCCG	ATCAGGCAAAACGCCACCAC	NM_001160226.1
*CNR2*	GGTGACAGAGATAGCCAATG	GCCAATGAACAGGTATGAGG	NM_001841.2
*NAPE-PLD*	CAGTAGAACAGTGTGTACGTAGAAG	CACTTCTAGAATGATACCCAAACTC	NM_001122838.1
*FAAH*	TATGAGACTGACAACTATACCATGC	CACGAAATCACCTTTGAAGTTCTGT	NM_001441.2
*DAGL*	GTGCCATCCGACATCATTGC	GCGGAGCATCTCTTGTGAAT	NM_006133.2
*MAGL*	ATGCAGAAAGACTACCCTGGGC	TTATTCCGAGAGAGCACGC	NM_001003794.2
*PPARα*	CTGGAAGCTTTGGCTTTACG	GTTGTGTGACATCCCGACAG	NM_001001928.2
*FASN*	CAGAGCAGCCATGGAGGAG	TAGAGCCCCGCCTTCCAG	NM_004104.4
*GLUT1*	TGGCATCAACGCTGTCTTCT	AGCCAATGGTGGCATACACA	NM_006516.2
*GLUT9*	TCCAGAGGGGCATGAAAACTC	CGAGCAGGACCAGTCCAATTT	NM_001001290.1

**Table 3:** Vibrational analysis of second derivative spectra of uFSH, rFSH and hMG samples. The wavenumbers of the most relevant biological absorptions are listed, together with the corresponding vibrational mode and biochemical assignment.

**Wavenumber**	**Vibrational Mode and Biochemical Assignment**	**References**
~2960	Asymmetric stretching vibration of CH_3_ groups (lipids)	[62,63]
~2925	Asymmetric stretching vibration of CH_2_ groups (lipids)	[62,63]
~1741, ~1716	Stretching vibration of C = O moieties (triglycerides and fatty acids)	[64,65]
~1655, ~1545	Amide I and II bands (proteins)	[62]
~1464, ~1398	Bending modes of methyl and methylene groups in lipids’ and proteins’ side chains	[66]
~1169	Stretching vibration of C-O moieties of carbohydrates	[66]
~1053	Stretching vibration of C-OH groups in carbohydrates	[67,68]
~1116	Symmetric stretching vibrations of P-O-C moieties	[69]
~1238, ~1088	Asymmetric and symmetric stretching vibrations of PO_2_- groups (phosphates)	[69]
